# Four-year follow-up of corneal aberrations and visual functions of myopic patients after laser in situ keratomileusis

**DOI:** 10.12669/pjms.316.8338

**Published:** 2015

**Authors:** Tai-Xiang Liu, Yong-Tao Chen, Ting-Ting Dan, Rong Shi, Shao-Rong Linghu, Hai-Xiang Li

**Affiliations:** 1 Tai-Xiang Liu, Guizhou Ophthalmic Hospital, the Affiliated Hospital of Zunyi Medical College, Zunyi 563003, Guizhou Province, China; 2 Yong-Tao Chen, Guizhou Ophthalmic Hospital, the Affiliated Hospital of Zunyi Medical College, Zunyi 563003, Guizhou Province, China; 3 Ting-Ting Dan, Guizhou Ophthalmic Hospital, the Affiliated Hospital of Zunyi Medical College, Zunyi 563003, Guizhou Province, China; 4 Rong Shi, Guizhou Ophthalmic Hospital, the Affiliated Hospital of Zunyi Medical College, Zunyi 563003, Guizhou Province, China; 5 Shao-Rong Linghu, Guizhou Ophthalmic Hospital, the Affiliated Hospital of Zunyi Medical College, Zunyi 563003, Guizhou Province, China; 6 Hai-Xiang Li, Guizhou Ophthalmic Hospital, the Affiliated Hospital of Zunyi Medical College, Zunyi 563003, Guizhou Province, China

**Keywords:** Myopia, LASIK, Corneal higher-order aberration, Visual function

## Abstract

**Objective:**

To report on 4-year follow-up of corneal higher-order aberrations and daily visual functions of myopic patients after laser in situ keratomileusis (LASIK).

**Methods:**

One hundred thirty four eyes of 67 patients who underwent LASIK guided by aspherical ablation were included in this study. The vision, corneal spherical aberration (SphA) and Coma were recorded before LASIK and at 6 month and 4 year after LASIK. The evaluation of the questionnaire about daily visual functions was performed by the same physician after LASIK.

**Results:**

No eye decreased the BCVA during 4 year follow-up. The effect index and safety index were 1.08±0.16, 1.11±0.17 and 1.12±0.16, 1.13±0.14 respectively at 6 month and 4 year post-LASIK. After LASIK the corneal SphA and Coma were significantly increased, however the difference between 6 month and 4 year post-LASIK was no statistical significance. Most patients (94.3%-92.4%) felt satisfaction or high satisfaction about the ability to perform each daily visual function after LASIK. Meanwhile there was still about 7.4%-9.2% patients who complained that they could not drive at night. Further analysis showed that the score of driving at night was negative correlation with corneal SphA (r=-0.645, *p*=0.040; r=-0.688, *p*=0.040 at 6 month and 4 year post-LASIK respectively).

**Conclusions:**

Our four-year follow-up outcomes indicated that the myopic patients after LASIK had the long-term stable corneal aberration and satisfaction of daily visual functions.

## INTRODUCTION

Myopia is world problem, and the Laser in Situ Keratomileusis (LASIK) as a main therapy for myopia had been used for over twenty years, which brought good therapeutic effect for myopic patients. However some patients even had acuity vision, they often complain that they had poor vision at night, especially the difficulty of driving at night.^1^,^2^ Previous study showed that the decrease of visual quality was correlation with the increased corneal higher-order aberrations due to LASIK.^3^,^4^ To decrease the aberrations and improve the visual quality, today several customized cutting pattern are performed.^5^-^7^ Unfortunately still there is no perfect effect about the corneal refractive surgery. In this present study we observed the corneal aberrations and visual functions of myopic patients after LASIK guided by aspherical ablation with 4-year follow-up to explore their long-term stability.

## METHODS

Sixty seven patients (32 men and 35 women, 134 eyes) who underwent LASIK guided by aspherical ablation at the Guizhou Ophthalmic Hospital of the Affiliated Hospital of Zunyi Medical College from March 2010 to March 2011 with 4 years follow-up after surgery were included in this study. The patient age at the time of surgery was (24.56±5.57) (range, 18 to 37) years. The preoperative manifest spherical equivalent (SE) was (-5.10±1.88) D (range, -1.25D~10.25D). Patients were excluded if they had corneal surgery, keratoconus, flap complications, eccentric ablation, epithelial in growt.

The routine examination about refractive surgery was performed. We used the Optikon-2000 and KeratronScout (Rome, Italy) to get the corneal topography. Every eye was checked 4 times, and then the best topography was selected to analyze the corneal higher-order aberrations. All these examinations were performed by one doctor. The LASIK procedure included mechanical flap preparation using Moria II microkeratome (Moria, Antony, France) with an intended 110-µm corneal flap. Excimer ablation guided by aspherical ablation for all eyes was performed using SCHWIND ORK Esiris excimer laser platform. An ablation zone of 6.5mm has been selected.

The refraction, uncorrected visual acuity (UCVA), best corrected visual acuity (BCVA), anterior corneal spherical aberration (SphA) and Coma were recorded before LASIK and at 6 month and 4 year after LASIK. The evaluation of the questionnaire^8^ about the daily visual functions was performed by the same physician after LASIK (Table-I)

**Table-I:** The evaluation of the daily visual functions after LASIK (x̄±s).

Visual function	6 Month Post-operation	48 Month Post-operation		
	mean	Mean	t	p
Reading in daylight	7.25± 1.29	7.75±2.11	-0.64	0.53
Reading in light	6.25±1.67	7.75±2.11	-1.77	0.10
Watching TV	7.25±1.29	8.25±1.05	-1.90	0.07
Watching movie	7.25±1.29	8.25±1.05	-1.90	0.07
Driving in daylight	7.50±1.34	8.25±1.05	-1.33	0.20
Driving at night	5.25±1.29	6.25±1.77	-1.42	0.17
Reading computer screen	7.25±1.29	7.25±2.11	0.00	1.00
Playing sports	7.75±1.29	8.25±1.05	-0.95	0.36
Swimming	6.25±1.67	7.75±2.11	-1.77	0.10
Shaving/Applying makeup	7.92±1.29	8.25±1.08	-0.56	0.58
Shopping	7.75±1.29	8.25±1.05	-0.95	0.36

The results are expressed as mean ± standard deviation (SD). All statistical analyses were performed using SPSS for Windows (SPSS, Inc, Chicago, IL, USA. version 17.0). The difference of corneal aberrations between pre-operation and post-operation were analyzed using analysis of variance. The score of visual functions between 6 month and 4 years after surgery was used for independent-sample t test to compare; and used Pearson correlation to analyze the correlation of the corneal aberration and the score of daily visual function after operation. A value of P<0.05 was considered statistically significance.

## RESULTS

### 1: The visual acuity after surgery

All eyes had 0.8 or more UCVA, and no eye lost 1 line or more in BCVA during 48 month period after treatment. The effect index and safety index were 1.08±0.16, 1.11±0.17 and 1.12±0.16, 1.13±0.14 respectively at 6 month and 4 year after LASIK. No significant difference between 6 month and 4 years post-LASIK about the effect index and safety index (Table-II).

**Table-II:** The effect index and safety index after LASIK (x̄±s).

Time	UCVA	BCVA	Effect index	Safety index
Pre-operation	0.08±0.02	1.14±0.17
6 Month Post-operation	1.21±0.12	1.26±0.22	1.08±0.16	1.11±0.17
48 Month Post-operation	1.26±0.13	1.28±0.20	1.12±0.16	1.13±0.14
t	-0.62	-0.24
P	0.54	0.81

### 2: Anterior corneal SphA and Coma

After LASIK the anterior corneal SphA and Coma were significantly increased, however the difference between 6 months and 4 years post-LASIK had no statistical significance (Table-II).

### 3: Visual functions

The response of negative or very negative about the daily visual function in questionnaire mean visual interference and the satisfaction or very satisfaction were considered as good visual function. There were 660 times and 737 times response about the daily visual functions at 6 month and 4 year post-LASIK respectively, and most patients (94.3% to 92.4%) felt satisfaction or high satisfaction about the ability to perform each visual function in daily activities after LASIK. However the reduction of night vision was the main complaint in our follow-up. Early follow-up, there had six patients (9.2%) felt negative about the driving at night. At 4 year after LASIK, 5 patients (7.4%) still had the same complaint. The difference of the score of visual function between 6 month and 4 year after operation had no statistical significance (Table-I). And the score of driving at night was negative correlation with corneal SphA (r=-0.645, *p*=0.040; r=-0.688, *p*=0.040 at 6 months and 4 years post-LASIK respectively, (Table-IV).

**Table-III:** Comparison of the corneal aberrations after operation (x̄±s).

Aberration	Pre-operation	6 Month Post-operation		48 Month Post-operation		
	mean	mean	p1	mean	p1	p2
Coma	0.21±0.10	0.56±0.14	0.00	0.44±0.25	0.00	0.25
SphA	0.33±0.12	0.77±0.33	0.00	0.65±0.08	0.00	0.21

***Note:*** p1 represented the comparison between pre-operation and post-operation about SphA and Coma; p2 represented the comparison between 6 month and 4 year post-LASIK about SphA and Coma.

**Table-IV:** The correlation of the corneal aberration and the daily visual function after operation.

Visual function	6 Month Post-operation		48 Month Post-operation	
	Coma	SphA	Coma	SphA
Reading in daylight	0.42	0.41	-0.08	0.36
Reading in light	0.03	0.02	-0.08	0.36
Watching TV	0.42	0.41	-0.08	0.36
Watching movie	0.42	0.41	-0.08	0.36
Driving in daylight	0.13	0.18	-0.08	0.36
Driving at night	-0.59	-0.66*	-0.25	-0.69*
Reading computer screen	0.42	0.41	-0.53	-0.02
Playing sports	-0.33	-0.19	0.35	0.01
Swimming	0.03	0.02	-0.08	-0.04
Shaving/Applying makeup	0.40	0.51	-0.08	0.36
Shopping	-0.33	-0.19	-0.08	0.36

Note:

* : P<0.05

## DISCUSSION

In the present study, we observed the long-term changes of anterior corneal SphA and Coma, and then evaluated the patients’ satisfaction about the daily visual functions after LASIK through questionnaire.

It was well known that the BCVA was the most important index to assess the safety after refractive operations.^9^ In our study all of eyes had 0.8 or more UCVA, and no loss(one line or mor)e in BCVA during 48 month period after treatment. This data indicated that myopic patients had long-term good effect and safety after LASIK.

Previous study showed that the third-order and fourth-order aberrations were the main component in human cornea,^10^,^11^ and SphA and Coma were the most common aberrations among them. In normal eyes, the anterior cornea SphA is positive, while the negative SphA of posterior cornea and lens could partially compensated it. So the higher-order aberrations of whole eye were lower than that of corneal aberrations. Research showed that the corneal aberrations were the main component in the whole higher-order aberrations. The refractive surgery changes the anterior corneal aspheric shape, so the corneal aberrations were increased significantly after surgery, as well as the aberrations of whole eye.^12^ Under the natural light, the diameter of pupil was about 2.5mm to 4.0mm, while under the dark environment it was 6.0mm or more.^13^ The ablative zone was 6.5mm in our LASIK procedure guided by aspherical ablation, so in present study we analyzed the anterior corneal SphA and Coma at 6.5mm zone. During 4-years follow-up, the data showed that SphA and Coma after LASIK were significantly increased. However we found that the difference between 6 month and 4 year post-LASIK had no statistical significance. It demonstrated that there had long-term stable corneal SphA and Coma after LASIK.

Though there are good long-term effect and safety, and we also found that most people (94.3% to 92.4%) felt satisfaction or high satisfaction about the ability to perform each visual function in daily activities after LASIK, there still some people complained the visual disturbance at night, especially driving at night. Early stage in our follow-up, 9.2% patients felt negative about the driving at night. Unfortunately, this complaint could not attenuate with time. Even at 4 year after LASIK, still 7.4% patients had the same complaint. However in our 4-year follow-up, no new patient had the same complaint. At six month and 4 year after LASIK, patients had the similar score about the daily visual function assessment. These indicated that at six month after surgery the visual function could satisfy with patients’ daily activities. Even with time, there had yet the stable evaluation about the ability to perform each function. We further analyzed the correlation of the score of visual function and the corneal aberrations. We found that the score of driving at night either at six months or 4 years post-LASIK was negative correlation with corneal SphA. And it was speculated that this phenomenon would not be improved for a long time. So it should be prudently selected to perform for myopic patients who work under high risk environment. However, the correlation of the higher-order aberrations of whole eye and the daily visual function and the longer follow-up should be further explored.
